# Towards Practical Application of *Verticillium isaacii* Vt305 to Control Verticillium Wilt of Cauliflower: Exploring Complementary Biocontrol Strategies

**DOI:** 10.3390/plants9111469

**Published:** 2020-10-30

**Authors:** Silke Deketelaere, Katrijn Spiessens, Sabien Pollet, Lien Tyvaert, Luc De Rooster, Danny Callens, Soraya C. França, Monica Höfte

**Affiliations:** 1 Laboratory of Phytopathology, Department of Plants and Crops, Faculty of Bioscience Engineering, Ghent University, Coupure Links 653, B-9000 Ghent, Belgium; silke.deketelaere@ugent.be (S.D.); schoonbaert-tyvaert@hotmail.com (L.T.); soraya.franca@biobestgroup.com (S.C.F.); 2 Research Station for Vegetable Production npo, Duffelsesteenweg 101, B-2860 Sint-Katelijne-Waver, Belgium; katrijn.spiessens@telenet.be (K.S.); luc.de.rooster@proefstation.be (L.D.R.); 3 Inagro vzw, Ieperseweg 87, B-8800 Rumbeke-Beitem, Belgium; sabien.pollet@inagro.be (S.P.); danny.callens@inagro.be (D.C.); 4 R&D Department, Biobest Group NV, Ilse Velden 18, B-2260 Westerlo, Belgium

**Keywords:** *Verticillium longisporum*, biological control, *Verticillium isaacii*, cauliflower, endophytes, soil-borne pathogen, green manure

## Abstract

Verticillium wilt is one of the most important diseases of cauliflower and can lead to serious economic losses. In this study, two complementary strategies were explored to employ the antagonistic capacity of *Verticillium isaacii* towards Verticillium wilt of cauliflower. The first strategy focused on introducing *V. isaacii* Vt305 by artificial inoculation of cauliflower plantlets at the nursery stage. Two inoculum types (spores and microsclerotia of *V. isaacii* Vt305) and different concentrations of microsclerotia were tested in greenhouse and field trials. Seed treatment with 500 microsclerotia seed^−1^ led to a satisfying biocontrol level of Verticillium wilt. In addition, the PHYTO-DRIP^®^ system was successful in delivering the microsclerotia to cauliflower seeds. The second strategy relied on the stimulation of the natural *V. isaacii* populations by rotating cauliflower with green manures and potato. Four green manure crops and potato were tested during multiple field experiments. Although these crops seemed to stimulate the *V. isaacii* soil population, this increase did not result in a control effect on Verticillium wilt of cauliflower in the short term. Importantly, our results indicate that the use of green manures is compatible with the application of *V. isaacii* Vt305 as biocontrol agent of Verticillium wilt in cauliflower.

## 1. Introduction

The soil-borne pathogen *Verticillium longisporum* (C. Stark) Karapapa, Bainbr. & Heale causes serious vascular disease in cauliflower (*Brassica oleracea* var. *botrytis* L.). The disease was first reported in coastal California (USA) in 1990 [1] and nearly 10 years later in Belgium [2]. Symptoms observed in *Verticillium*-infested fields are asymmetric chlorosis of the leaves, wilting, stunted growth, and vascular discoloration of the roots and stem [1,2]. Cauliflower is one of the most important field vegetables in Belgium with a production surface area of more than 5700 ha in 2020 [3]. Verticillium wilt can lead to serious economic losses in cauliflower as both the quality and yield of the heads are reduced and is therefore a major threat to cauliflower production in Belgium.

The application of biocontrol agents might be a valuable and ecofriendly control strategy of Verticillium wilt that conforms to the integrated pest management (IPM) approach [4]. Only few bacterial isolates with biocontrol activity against *V. longisporum* in oilseed rape have been reported [5,6,7,8].

França et al. observed a negative correlation between Verticillium wilt and the presence of *Verticillium tricorpus*-like organisms in a Belgian cauliflower field [9]. Although these organisms morphologically resemble *Verticillium tricorpus* I. Isaac, phylogenetic analysis revealed that at least some of them belong to *Verticillium isaacii* Inderb. et al. [10]. In fact, the isolate Vt305, obtained from this suppressive cauliflower field in Belgium, was identified as *V. isaacii* [9]. Further research in controlled conditions has shown that Vt305 behaves as endophyte in cauliflower plants and can protect them against Verticillium wilt [11]. These results indicated that *V. isaacii* Vt305 is a promising biocontrol agent of Verticillium wilt of cauliflower.

Towards practical application of putative beneficial *V. isaacii*, two complementary biocontrol strategies were explored in this study. The first strategy was based on introducing *V. isaacii* Vt305 by artificial inoculation of cauliflower plantlets at the nursery stage. The second strategy relied on the stimulation of the natural *V. isaacii* populations by rotating cauliflower with green manures and potato. França et al. found that rotation of cauliflower with potato increased the density of *V. tricorpus*-like organisms in the Verticillium wilt-suppressive cauliflower field [9]. Interestingly, it has also been reported that cropping of *Verticillium*-resistant potato clones was accompanied by increases of bacterial antagonists and moreover resulted in 8–50 fold increases of *V. tricorpus* compared to a susceptible potato cultivar [12]. Use of green manure crops has been considered as an important practice in agroecological farming systems. Green manures offer substantial benefits to the soil, including increased organic matter and nutrients, improved soil structure, and weed and erosion control [13]. The strategy of using green manures to manipulate the indigenous soil microbial community in an attempt to achieve disease control is gaining increased interest. Increasing the density and diversity of pathogen-inhibitory microorganisms, such as fluorescent *Pseudomonas*, Streptomycetes and non-pathogenic *Fusarium* spp. through the incorporation of green manures has been shown in several studies [14,15,16,17]. The green manure crops tested in this study are already frequently used in the rotation system of cauliflower, except for sticky nightshade. Ryegrass (*Lolium* spp. L.) has been recommended as a lignin-rich amendment to reduce *Verticillium* density in soil [2,9]. *Phacelia tanacetifolia* Benth, member of the Boraginaceae Juss. plant family, is a popular green manure crop in rotations with vegetable crops and is considered as a good catch crop to reduce nitrogen leaching. Phacelia has been proposed as green manure crop to reduce Verticillium wilt of hop plants [18] and it is therefore interesting to test this crop in rotation with cauliflower. Sticky nightshade (*Solanum sisymbriifolium* Lam.) has been suggested as a trap crop for potato cyst nematodes [19]. Since this nematode is also a problem in Belgian fields and cauliflower is often rotated with potato, *S. sisymbriifolium* is an interesting green manure crop in our cauliflower fields. Black oat (*Avena strigosa* Schreb.) is a rapid growing leafy cereal crop and therefore very useful to suppress weeds. As a cover crop, black oat has been shown to resist or inhibit root-knot nematodes [20]. Because of these characteristics, black oat is commonly used as green manure crop in Belgium.

The present study aimed to evaluate the capacity of *V. isaacii* Vt305 to protect cauliflower in greenhouse and field conditions. Two inoculum types (spores and microsclerotia of *V. isaacii* Vt305) and different doses of microsclerotia were applied on cauliflower seedlings. In addition, the PHYTO-DRIP^®^ system was evaluated for microsclerotia application on seeds at nursery. A second objective of this study was to investigate the effect of green manure crops (ryegrass, Phacelia, *Solanum sisymbriifolium* and black oat) and potato on the *Verticillium* microsclerotia densities in soil and the consequence of potential changes in *Verticillium* soil populations on Verticillium wilt in cauliflower. Finally, the combined effect of green manure crops and artificial inoculation of cauliflower with *V. isaacii* Vt305 on Verticillium wilt of cauliflower was investigated.

## 2. Results

### 2.1. Production of V. isaacii Vt305 Microsclerotia

The production of microsclerotia on modified soil extract agar covered with a cellophane disc was found to be a fast and consistent production method on lab scale. Huge amounts of microsclerotia were formed per plate after 14 days of incubation in the dark. On average this method yielded 8466.7 ± 472.6 microsclerotia mg^−1^ powder corresponding to (8.5 ± 0.5) × 10^6^ microsclerotia per plate. Importantly, microsclerotia could be collected separately, which allows a correct dosing.

### 2.2. Efficacy of V. isaacii Vt305 in Greenhouse Conditions

Two greenhouse experiments were carried out to assess the effect of *V. isaacii* Vt305 against Verticillium wilt in soil naturally infested with *V. longisporum. V. isaacii* Vt305 was applied as spores or as microsclerotia. Inoculation with spores was done by dipping the potting soil blocks of four-week-old plantlets in a spore suspension (10^6^ spores mL^−1^) 10 days before transplantation to the naturally infested soil. Microsclerotia (MS) suspensions were applied onto the cauliflower seeds in different concentrations (100 MS, 500 MS, and 1000 MS) and 30 days later plants were transplanted to the naturally infested soil (2974 fg *V. longisporum* DNA g^−1^ soil and 245 fg *V. isaacii* DNA g^−1^ soil).

**Figure 1:**
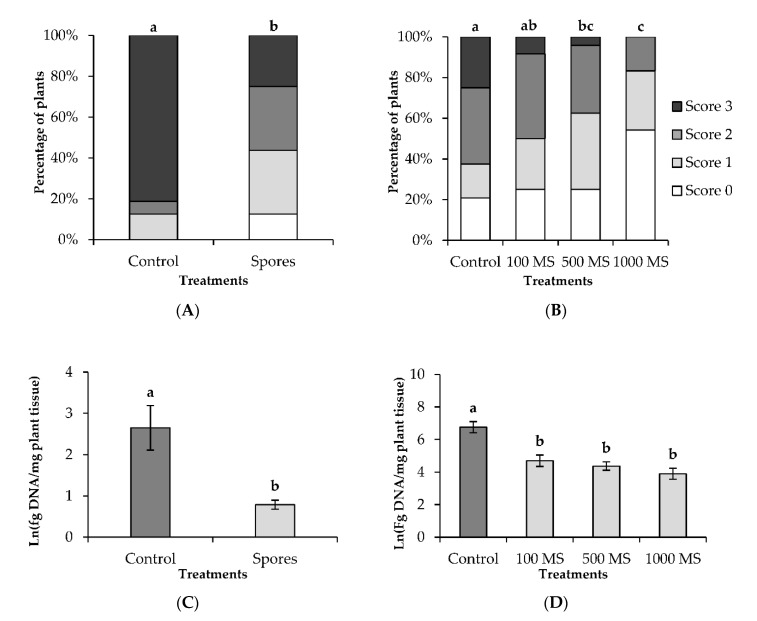
Biocontrol of Verticillium wilt in cauliflower in greenhouse conditions when *V. isaacii* Vt305 was applied as spores or as microsclerotia (MS). (**A**) Vascular discoloration 70 days post transplantation (dpt) to naturally infested soil of cauliflower plants inoculated with *V. isaacii* Vt305 via root dip in a spore suspension with a concentration of 10^6^ spores mL^−1^ or mock-inoculated via root dip in sterile water in case of the control; (**B**) Vascular discoloration 103 dpt to naturally infested soil of cauliflower plants inoculated with *V. isaacii* Vt305 via seed treatment with a microsclerotia suspension applied in different concentrations (100, 500, or 1000 microsclerotia plant^−1^) or mock-inoculated via seed treatment with sterile water in case of the control. Results on vascular discoloration are shown as percentage of plants (*n* = 12) with a specific score for vascular discoloration: 0 = no vascular discoloration; 1 = vascular discoloration of <50% of stem length; 2 = vascular discoloration of 50–75% of stem length; and 3 = vascular discoloration of 76–100% of stem length. (**C**) Amount of detected *V. longisporum* DNA in the hypocotyl 28 dpt when *V. isaacii* Vt305 was applied as spores; (**D**) Amount of detected *V. longisporum* DNA in the hypocotyl 103 dpt when *V. isaacii* Vt305 was applied as microsclerotia. Values are means ± standard error (*n* = 6). Colonization data were ln transformed before statistics. Bars indicated with the same letter are not significantly different according to a Mann–Whitney U-test in case of the vascular discoloration and according to a Tukey test in case of the colonization data (*p* < 0.05).

Dipping the cauliflower plantlets in a spore suspension of *V. isaacii* Vt305 significantly suppressed disease symptoms of Verticillium wilt in cauliflower compared to the untreated control (Figure 1A): 25% of the cauliflower plants showed severe vascular discoloration (score 3), while 80% of the untreated control plants received a score of 3.

Prior to transplantation, the hypocotyls of the cauliflower plantlets inoculated with microsclerotia were sampled to monitor the colonization by *V. isaacii* Vt305. *V. isaacii* DNA was detected in the hypocotyls of all sampled plants when a dose of 100 MS per seed was applied. At doses of 500 MS and 1000 MS, *V. isaacii* was present at detectable levels in the hypocotyls of four out of six plants. For all treatments only small amounts of *V. isaacii* DNA were detected, which indicates that 30 days after sowing *V. isaacii* Vt305 could not colonize the hypocotyl to a high extent.

Pre-inoculation with *V. isaacii* Vt305 microsclerotia clearly reduced the vascular discoloration of the stem (Figure 1B). This reduction was statistically significant when seeds were treated with 500 MS or 1000 MS. Vascular discoloration was prevented in more than half of the stems when 1000 MS was applied. Moreover, a dose-effect was observed; a higher dose of *V. isaacii* Vt305 microsclerotia resulted in lower scores for vascular discoloration of the stems.

The colonization of the hypocotyl by *V. longisporum* was significantly reduced by *V. isaacii* Vt305 at 28 days post transplantation (dpt) to naturally infested soil when the biocontrol agent was applied as spores (Figure 1C). The colonization of the cauliflower plants by *V. longisporum* was quantified at different time points during the greenhouse experiment in which microsclerotia were used as inoculum of *V. isaacii* Vt305. No significant differences in *V. longisporum* colonization were detected in roots and hypocotyls between control plants and inoculated plants at 33 dpt and 68 dpt. At harvest (103 dpt) a significant lower amount of *V. longisporum* DNA was found in the hypocotyl of plants inoculated with *V. isaacii* Vt305 (Figure 1D), while comparable levels were detected in the roots of all plants. Colonization by *V. longisporum* did not differ much between the plants treated with different concentrations of the *V. isaacii* Vt305 microsclerotia suspension.

In both greenhouse experiments, *V. isaacii* was also detected in the roots and hypocotyls of the control plants after transplantation in naturally infested soil. The amount of *V. isaacii* present in the naturally infested soil (245 fg DNA g^−1^ soil) was sufficient to colonize the control plants, but did not offer protection against Verticillium wilt (Figure 1). It was furthermore remarkable that at time points close to harvest, the hypocotyls of control plants were colonized to a higher extent by *V. isaacii* species than the hypocotyls of plants pre-inoculated with *V. isaacii* Vt305 (data not shown).

### 2.3. Efficacy of V. isaacii Vt305 in Field Conditions

#### 2.3.1. Application of Different Doses of *V. isaacii* Vt305 Microsclerotia at Three Locations in Three Different Cauliflower Cultivars

For the field experiments, cauliflower seeds were manually dripped with a microsclerotia suspension of *V. isaacii* Vt305 or with tap water (control). After growing the plants for four weeks in the nurseries, they were planted in naturally infested fields. At harvest, the vascular discoloration of cauliflower stems was scored and a disease index was calculated. The quality of the cauliflower curds was also evaluated to determine the percentage of marketable curds.

The effect of applying different concentrations of *V. isaacii* Vt305 microsclerotia on Verticillium wilt was tested during two field experiments at locations in Ardooie (cultivar Clapton) and Bornem (cultivar Korlanu) in 2014. Seed treatment with microsclerotia significantly reduced the disease index in Ardooie regardless of the dose (Table 1). When treated with *V. isaacii* Vt305, more than half of the plants showed no or minor vascular discoloration. The main differences between treatments were found in the number of plants with severe vascular discoloration (score of 3). On average, 24.5% of the control plants were given a score of 3, while after treating the seeds with 300 MS of *V. isaacii* Vt305 only 11.1% of the plants received a score of 3. When doses of 500 MS or 1000 MS were applied, no stems or only 2.2% of the stems showed severe vascular discoloration, respectively. In practice, cauliflower plants with scores of 1 and 2 for vascular discoloration still produce marketable curds in most cases. Only 75.6% of the evaluated control plants produced curds that met the market standards in the experiment in Ardooie. After applying *V. isaacii* Vt305 the number of marketable cauliflower curds was significantly higher and there were almost no yield losses when seeds were treated with 500 and 1000 MS. There was no significant effect of *V. isaacii* Vt305 application on the disease index in Bornem, although a dose of 500 MS tended to reduce the disease index compared to the control treatment (Table 1). This could be explained by the significant increase of the number of healthy plants after pre-inoculation with a dose of 500 MS. Furthermore, it is important to notice that none of the evaluated plants showed severe symptoms (score of 3) and no loss of marketable curds occurred. The low disease pressure possibly masked the protective effect of *V. isaacii* Vt305.

During the field experiment in Oppuurs in 2015, cauliflower seeds of the cultivars Korlanu and Clarina were treated with a microsclerotia suspension of *V. isaacii* Vt305 at a rate of 500 MS per seed. This concentration of microsclerotia was chosen because it led to satisfying biocontrol of Verticillium wilt in the field experiments carried out in 2014. Application of *V. isaacii* Vt305 resulted in more plants with scores of 0 or 1 for vascular discoloration for both cultivars (Table 1), but significant differences were found only in the cultivar Clarina.

#### 2.3.2. Application of *V. isaacii* Vt305 Microsclerotia with the PHYTO-DRIP^®^ System

Cauliflower seeds of the cultivar Clapton with different coatings were inoculated with *V. isaacii* Vt305 microsclerotia using the PHYTO-DRIP^®^ system (Syngenta, Basel, Switzerland). With this system a single drop of the microsclerotia suspension was dripped onto each seed during the seeding process resulting in a dose of 360 MS per seed. Seeds of the control plants were dripped with tap water. The use of the PHYTO-DRIP^®^ system resulted in easy and fast application of the *V. isaacii* Vt305 microsclerotia suspension on the cauliflower seeds. The microsclerotia did not obstruct the filters of the system and no other difficulties were observed. Four-week-old plantlets were subsequently planted in a field with a history of Verticillium wilt of cauliflower located in Ardooie. Table 2 shows that the seed treatment with *V. isaacii* Vt305 microsclerotia protected cauliflower against Verticillium wilt. At harvest (98 dpt), more than 90% of the plants pre-inoculated with *V. isaacii* Vt305 were scored 0 or 1. Control plants showed more severe vascular discoloration and most plants received a score of 2 or 3. These differences in disease severity are illustrated by the disease indexes, which were more than 50% lower when the seeds were treated with *V. isaacii* Vt305. No loss of marketable curds was found in plants inoculated with *V. isaacii* Vt305. Importantly, the conventional seed coatings with fungicides did not influence the biocontrol effect of *V. isaacii* Vt305 against Verticillium wilt in cauliflower.

### 2.4. Effect of Potato and Green Manure Crops on Verticillium Soil Populations and Verticillium Wilt of Cauliflower

#### 2.4.1. Colonization of Green Manure Crop Residues by *V. isaacii*, *V. longisporum*, and *V. dahliae*

By determining the colonization of the crop residues of Phacelia, ryegrass, sticky nightshade, and black oat by *V. isaacii*, *V. longisporum* and *V. dahliae*, we aimed to estimate their contribution to the build-up of soil inoculum of these three *Verticillium* species. After growing the crops in naturally infested soil, fragments of these crops were put in nylon bags and buried in the soil to mimic incorporation in the field. *V. isaacii* and *V. dahliae* were detected in the crop residues of all tested crops (Table 3). In particular, the residues of ryegrass were highly colonized by *V. isaacii* and those of sticky nightshade by *V. dahliae*. A detectable colonization by *V. longisporum* of the crop residues was only found for black oat. These results indicated that incorporation of Phacelia, ryegrass, sticky nightshade, and black oat might increase the soil inoculum of *V. isaacii* and *V. dahliae*. Importantly, there were no indications that the tested crops, apart from black oat, will stimulate *V. longisporum* in the soil.

#### 2.4.2. Effect of Potato and Green Manure Crops on Soil Populations of *Verticillium* spp. and the Impact on Verticillium Wilt in Cauliflower

The effect of potato, Phacelia, ryegrass, sticky nightshade, and black oat on the soil populations of *V. isaacii*, *V. longisporum*, and *V. dahliae* and on Verticillium wilt in the following crop cauliflower was tested during field experiments in 2013 and 2014 at different locations in Ardooie and Puurs. Plots left fallow before the cultivation of cauliflower were used as control.

**Figure A1:**
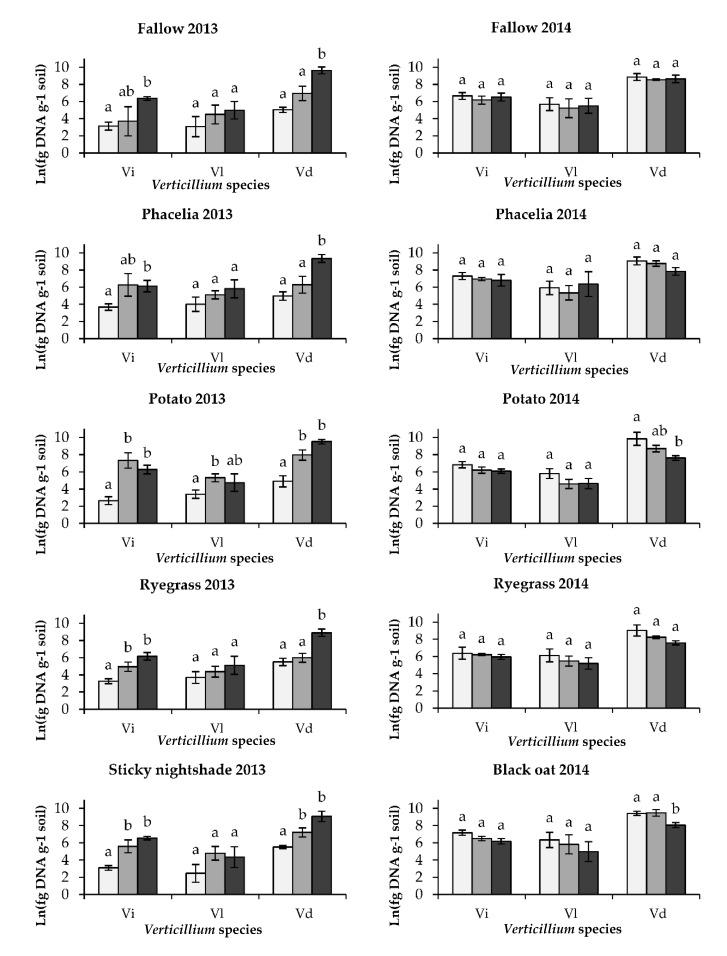
Inoculum densities of *V. isaacii* (Vi), *V. longisporum* (Vl) and *V. dahliae* (Vd) at three time points (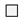: sowing/planting of crop 1, 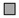: incorporation of crop 1 and 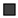: harvest of cauliflower) in a grower’s field located in Ardooie during the growing seasons of 2013 and 2014 in five cover crop systems. Values are the mean of 4 replicates and the vertical bars represent the standard error of the mean. Data were ln transformed before statistics. For each *Verticillium* species, bars with different letters indicate statistical differences according to Tukey tests (*p* < 0.05).

**Figure A2:**
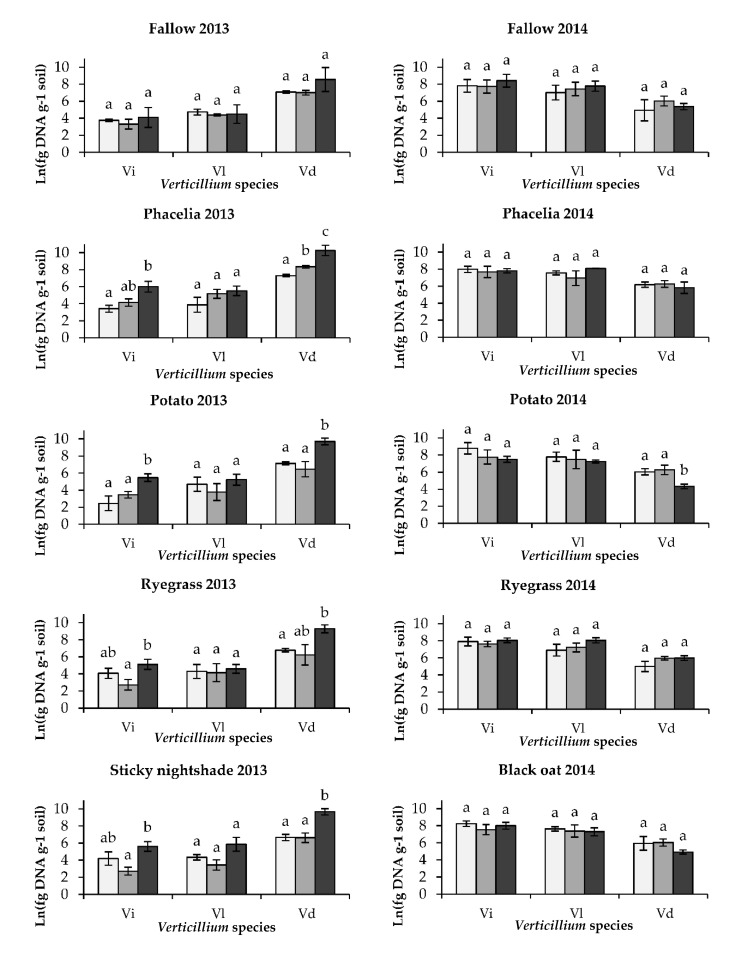
Inoculum densities of *V. isaacii* (Vi), *V. longisporum* (Vl) and *V. dahliae* (Vd) at three time points (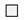: sowing/planting of crop 1, 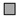: incorporation of crop 1 and 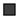: harvest of cauliflower) in a grower’s fields located in Puurs during the growing seasons of 2013 and 2014 in five cover crop systems. Values are the mean of 4 replicates and the vertical bars represent the standard error of the mean. Data were ln transformed before statistics. For each *Verticillium* species, bars with different letters indicate statistical differences according to Tukey tests (*p* < 0.05).

The inoculum densities of *V. isaacii*, *V. longisporum* and *V. dahliae* on the different time points are shown per location for both years in Figure A1 and Figure A2. During the field experiment in Ardooie in 2013, a significant increase of the *V. isaacii* population was detected for all five cover crop systems and the largest increase was observed when potato preceded cauliflower. In the same year, the increase in the soil population of *V. isaacii* was significant after Phacelia and potato in the field in Puurs. The level of *V. longisporum* soil inoculum did not change significantly during the field experiments in 2013 for all five cover crop systems. In 2013, a significant increase of the *V. dahliae* population was observed in the field in Ardooie, irrespective of the cover crop system. The level of *V. dahliae* also increased over time in Puurs. Here, the increase was statistically significant when a cover crop preceded the cultivation of cauliflower.

Concerning the field experiments carried out in 2014, it was remarkable that the initial inoculum densities of all three *Verticillium* species were already quite high compared to 2013. The soil population of *V. isaacii* did not change much in all five cover crop systems during the field experiments in Ardooie and Puurs in 2014. In Ardooie, a decreasing trend was observed in the inoculum density of *V. longisporum* when ryegrass, black oat, or potato were cultivated before cauliflower. No big changes in the level of *V. longisporum* were detected in Puurs. In both fields, the *V. dahliae* inoculum density remained more or less stable when plots were left fallow before cultivating cauliflower. When a green manure crop or potato preceded cauliflower, a rather decreasing trend was observed regarding the *V. dahliae* population. Surprisingly, the soil population of *V. dahliae* significantly decreased after potato in the fields of Ardooie and Puurs. This was unexpected because potato is a host plant of *V. dahliae*. In addition, when black oat was used as a cover crop, the decrease of *V. dahliae* was statistically significant in the field in Ardooie.

**Figure 2:**
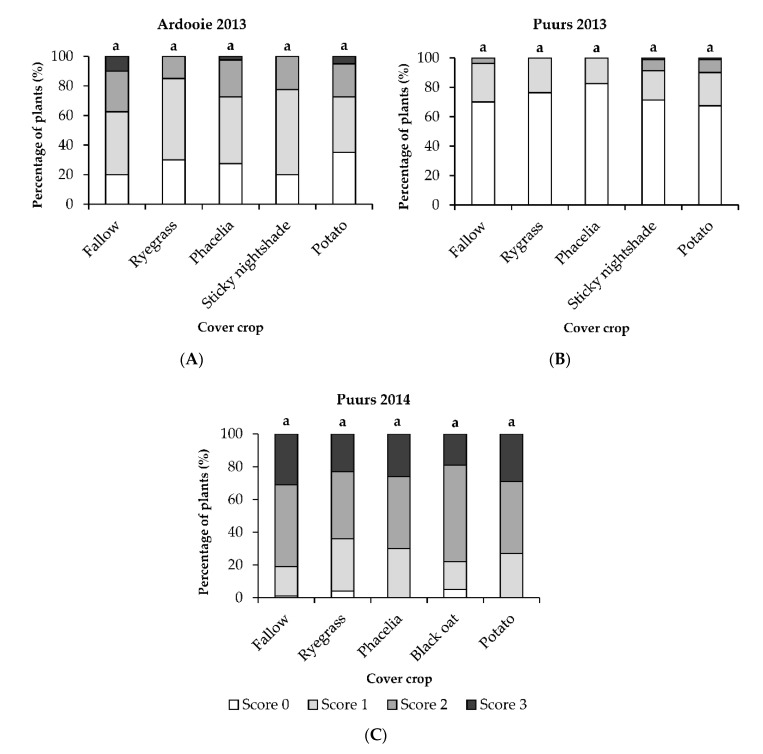
Verticillium wilt of cauliflower in grower’s fields located in Ardooie and Puurs during the growing seasons of 2013 and 2014 in five cover crop systems. (**A**) Vascular discoloration of the cauliflower stems in the field at Ardooie in 2013; (**B**) Vascular discoloration of the cauliflower stems in the field at Puurs in 2013; (**C**) Vascular discoloration of the cauliflower stems in the field at Puurs in 2014. Data are shown as the percentage of plants (*n* = 20) with a specific score for vascular discoloration at harvest of the cauliflower curds: 0 = no vascular discoloration; 1 = vascular discoloration of <50% of stem length; 2 = vascular discoloration of 50–75% of stem length; and 3 = vascular discoloration of 76–100% of stem length. Bars indicated with the same letter are not significantly different according to a Mann–Whitney U-test (*p* < 0.05).

After harvest of the curds, the vascular discoloration of the cauliflower stems was scored on a scale of 0 to 3 (Figure 2). In the first year (2013) of the field experiment in Ardooie, cauliflower stems exhibited less vascular discoloration after the tested crops compared to fallow. This reducing effect on Verticillium wilt was, however, not significant. Due to a severe clubroot infestation, it was not possible to score the vascular discoloration of the stems from the field in Ardooie in 2014. The disease pressure was low in the field of Puurs during the experiment in 2013 and no significant differences in disease severity of Verticillium wilt of cauliflower were observed between the five cropping systems. During the field experiment in Puurs in 2014, the disease pressure was quite high and only ryegrass and Phacelia seemed to lower Verticillium wilt compared to fallow. However, these effects were again not statistically significant. In the short term, the tested crops did not have a significant effect on Verticillium wilt of cauliflower. To determine the long term effects of these cropping systems field experiments over several years need to be carried out.

### 2.5. Combined Effect of Green Manure Crops and Treatment of the Cauliflower Seeds with V. isaacii Vt305 on Verticillium Wilt of Cauliflower

**Figure 3:**
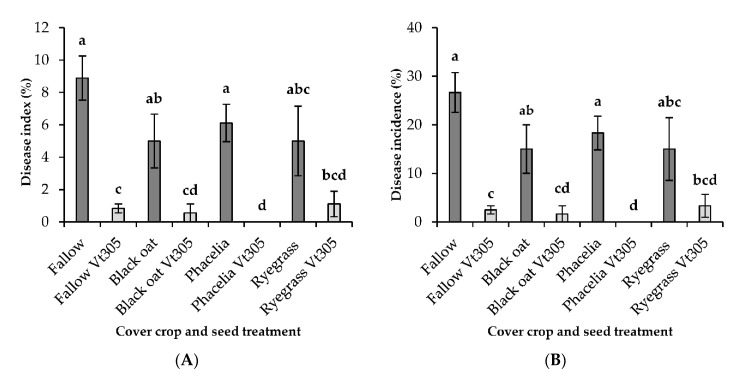
Effect of treatment with *V. isaacii* Vt305 on Verticillium wilt of cauliflower in a grower’s field in Puurs during the growing season of 2015 in four cover crop systems. Cauliflower seeds (cultivar Clarina) were treated with tap water or a microsclerotia (MS) suspension (500 MS per seed) indicated with ‘Vt305′. (**A**) Disease index of the cauliflower plants at harvest. The disease index was calculated based on the scores for vascular discoloration via the formula of Townsend–Heuberger [(0*a + 1*b + 2*c + 3*d)/(e*f)]*100; letters a, b, c, and d, are the numbers of plants for each disease score, the letter e is the highest score (=3), and f represents the number of observations (= 20). (**B**) Disease incidence of Verticillium wilt. Values are the mean of 4 replicates and the vertical bars represent the standard error of the mean. Bars indicated with the same letter are not significantly different according to a Mann–Whitney test (*p* < 0.05).

**Figure A3:**
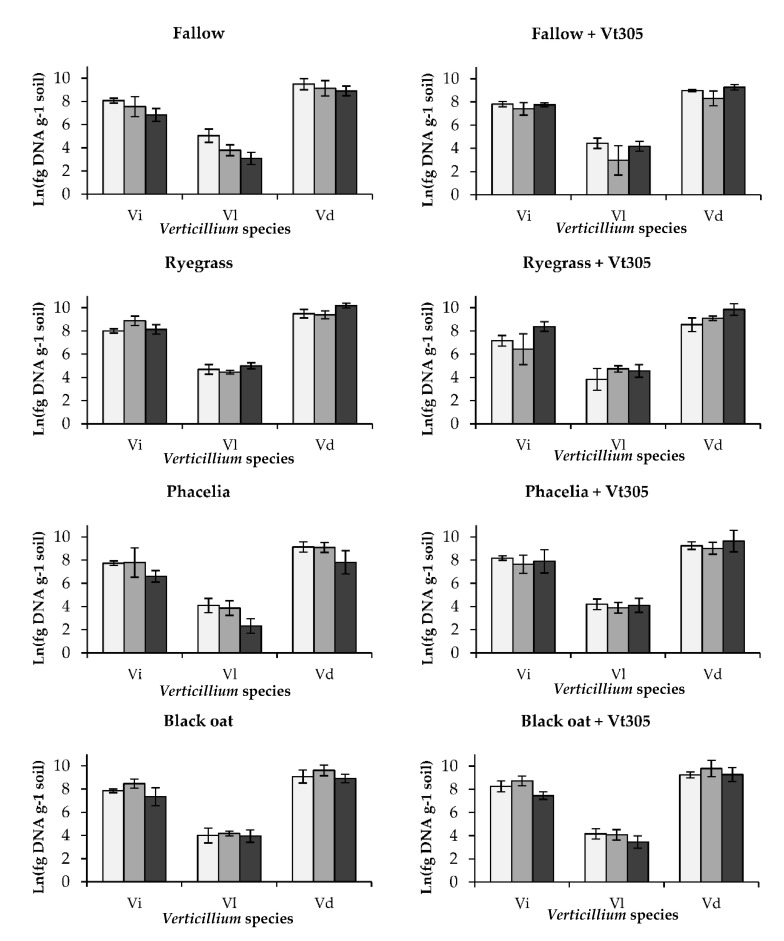
Inoculum densities of *V. isaacii* (Vi), *V. longisporum* (Vl) and *V. dahliae* (Vd) at three time points (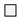: sowing/planting of crop 1, 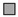: incorporation of crop 1 and 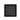: harvest of cauliflower) in a grower’s field located in Puurs during the growing season of 2015 in four cover crop systems and two treatment systems of the cauliflower seeds (left panel: no seed treatment; right panel: seed treatment with *V. isaacii* Vt305 microsclerotia indicated by ‘+Vt305’). Cauliflower seeds were treated with 0.5 mL of tap water (control plants) or 0.5 mL of a microsclerotia (MS) suspension of *V. isaacii* Vt305 resulting in 500 MS per seed. Values are the mean of 4 replicates and the vertical bars represent the standard error of the mean. Data were ln transformed before statistics. No statistical differences were detected for the different *Verticillium* species in the different growing systems using Tukey tests (*p* < 0.05).

The effect of combining green manure crops and seed treatment of cauliflower with *V. isaacii* Vt305 on Verticillium wilt was tested during a field experiment in a grower’s field located in Puurs. A dose of 500 microsclerotia per seed was used to treat the cauliflower seeds. At harvest of the cauliflower curds, vascular discoloration of the stems was evaluated. The percentage of affected plants and the disease index (disease severity) are shown in Figure 3. The cultivation of black oat, Phacelia, or ryegrass alone did not significantly reduce Verticillium wilt compared to the fallow plots. Pre-inoculation of the cauliflower plants with *V. isaacii* Vt305, however, resulted in a significant reduction of the disease severity and incidence in all four cover crop systems. The combination of Phacelia and seed treatment with *V. isaacii* Vt305 of cauliflower resulted in the best protection against Verticillium wilt as no vascular discoloration was observed in the evaluated cauliflower stems. The artificial inoculation with *V. isaacii* Vt305 was clearly the determining factor in the reduction of Verticillium wilt. The soil of the different plots was also sampled to determine the inoculum densities of *V. isaacii*, *V. longisporum*, and *V. dahliae* at different time points (Figure A3). In this way, we could determine if differences in Verticillium disease were the result of changes in the ratio between soil populations of *V. isaacii* and *V. longisporum*. However, the results of the soil analyses did not correlate with the observed disease pressure of *V. longisporum* in the different plots. The high variability in disease pressure between the different plots could not be linked to differences in the soil populations of *V. isaacii* and *V. longisporum*.

## 3. Discussion

The ability of *V. isaacii* Vt305 to protect cauliflower plants against Verticillium wilt in controlled conditions has previously been shown by Tyvaert et al. [11]. In the present work, we have demonstrated that *V. isaacii* Vt305 could also reduce Verticillium wilt of cauliflower in greenhouse and field conditions. Biocontrol of pathogenic *Verticillium* spp. by non-pathogenic *Verticillium* isolates has already been shown in cotton [21,22], potato [23,24], tomato [25,26], and lettuce [27]. The protection of plants against virulent *Verticillium* species by a non-pathogenic relative *Verticillium* species has been described as cross-protection. Induced resistance, competition for space and nutrients, and plant growth promotion are suggested to be involved in this phenomenon [26,28].

The capacity of *V. isaacii* isolates to form different structures is an interesting characteristic of a biocontrol agent, as this can be useful for application in different systems. Besides the production of conidia, the formation of three different survival structures has been reported for *V. isaacii* isolates [10]. We have shown that both dipping cauliflower plantlets in a conidial suspension of *V. isaacii* Vt305 and treating the seeds with microsclerotia of *V. isaacii* Vt305 reduced Verticillium wilt. However, we believe that microsclerotia are more interesting structures for practical applications than conidia. These robust surviving structures resist unfavorable conditions and remain viable for a long time [29], which are desirable traits for future formulation and application in practice. Because of the persistence of the microsclerotia in the soil, it is crucial to verify that *V. isaacii* Vt305 is not pathogenic for a wide variety of plants. Via artificial inoculation trials we could already confirm that *V. isaacii* Vt305 does not cause either vascular discoloration or other symptoms of Verticillium wilt in potato, chrysanthemum, oilseed rape, broccoli, pepper, tomato, hop, Phacelia, and sticky nightshade (unpublished results).

Early application of biocontrol isolates, before pathogen infection, has been reported to result in better protection [21,24,27]. Application of *V. isaacii* Vt305 microsclerotia to the seed delivers the biocontrol agent in close vicinity to the plant roots and allows early colonization of the seedlings. Inoculating seeds with microsclerotia of *V. isaacii* Vt305 using the PHYTO-DRIP^®^ system was compatible with the production system and quality requirements of plant nurseries producing cauliflower plantlets. Moreover, treating the seeds with a suspension of *V. isaacii* Vt305 microsclerotia was compatible with the conventional coatings of the cauliflower seeds with fungicides and insecticides. However, the tested seed coatings with thiram and iprodione are currently no longer approved by the EU and additional experiments will be necessary if new coatings are used. From a commercial point of view, it is important to produce the greatest quantity of viable propagules with the best quality as cheaply as possible [30]. We have evidence that *V. isaacii* microsclerotia can be produced on a large scale in a cost-effective manner using solid-state fermentation. When developing the formulation, it is important that the final product is convenient to use, safe to handle and have adequate shelf life.

In the field experiments, *V. isaacii* Vt305 reduced Verticillium wilt significantly in the cauliflower cultivars Clapton and Clarina, but not in the cultivar Korlanu (Table 1 and Table 2, Figure 3). These results might indicate an effect of the cultivar on the success of *V. isaacii* Vt305 to control Verticillium wilt in cauliflower. A cultivar effect might be due to differences in susceptibility to *V. longisporum*. It is plausible that in very susceptible cauliflower cultivars a significant reduction of Verticillium wilt is more difficult to achieve than in more resistant cultivars. Debode et al [31] have observed clear differences in susceptibility to *V. longisporum* among European cauliflower cultivars. However, data from cauliflower cultivar trials, carried out by the research stations Inagro and PSKW (Belgium), showed a comparable susceptibility to *V. longisporum* of the cultivars tested in our study. The different levels of biocontrol observed in the various cauliflower cultivars might also be the result of differences in degree of colonization by *V. isaacii* Vt305 of the cultivars or the result of cultivar-specific responses involved in the biocontrol. Hence, it is essential for the practical application of *V. isaacii* Vt305 to evaluate more (commercial) cauliflower cultivars, regarding their colonization by this biocontrol isolate.

**Figure A4:**
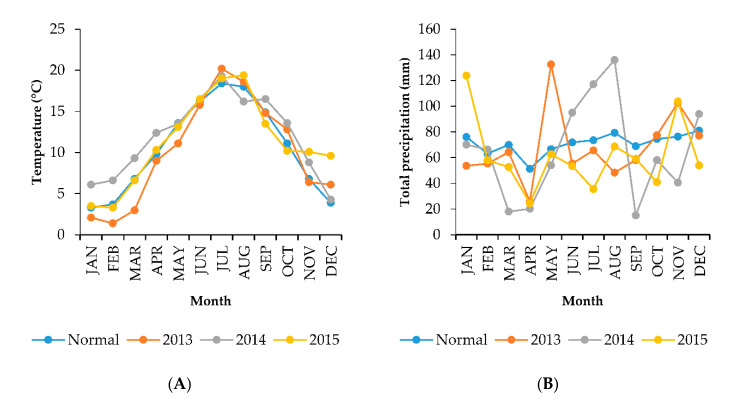
Meteorological data measured in Ukkel in 2013, 2014 and 2015. (**A**) Mean monthly temperature; (**B**) Total monthly precipitation [47].

In the field trials in 2013 regarding the effect of potato and green manure crops on *Verticillium* spp. densities in the soil, a significant increase of the *V. isaacii* soil population was observed in all five cover crop systems at both locations, except for the fallow treatment in the field of Puurs. This was in contrast with the results in 2014, where in none of the crop systems a significant increase was detected. Remarkably, the initial levels of *V. isaacii* in the different plots were much lower in 2013 compared to these in 2014. This might suggest that the extent of increase in soil population of *V. isaacii* depends on the initial density. In line with our results, Wiggins and Kinkel showed that the enrichment in streptomycete densities was density-dependent [16]. Soils with relatively low streptomycete densities had greater increases in streptomycete densities following incorporation of green manure crops than soil with relatively high streptomycete densities. The development and viability of *Verticillium* microsclerotia may be influenced by environmental factors, such as temperature, humidity, and microbial populations in the soil [32,33,34]. Interannual differences in the *Verticillium* inoculum densities observed in our field experiments might therefore also be due to different weather conditions in 2013, 2014, and 2015 (Figure A4). Compared to the field experiments in 2014 and 2015, the initial level of *Verticillium* soil inoculum was low in the field experiments in 2013 (Figure A1, Figure A2 and Figure A3). This observation might be explained by the low temperatures during the months February and March in 2013. The maximum temperature in March 2013 (6.1 °C) was even a negative record of the last 30 years. These low temperatures might have negatively affected the formation of microsclerotia in the crop residues. In addition, the release of *Verticillium* microsclerotia in the soil might have been reduced as a result of low microbial activity at these low temperatures in 2013.

Because fluctuations in the Verticillium populations were also noticed in the fallow treatments, it was difficult to draw conclusions about the effect of the crop systems on population densities of the three *Verticillium* species. Seasonal fluctuation of *Verticillium* microsclerotia density in soil was observed in cauliflower fields in Belgium. Peaks in *Verticillium* microsclerotia in spring and summer were followed by drop-offs in autumn [9]. In our study, we did not observe such seasonal fluctuation. The use of a different detection method (qPCR instead of a plating technique) might partly account for this. In addition, the sequence of crops and the timing of cultural practices differed between both studies. These factors might influence the formation, release from crop residues and germination of *Verticillium* microsclerotia and thus the amount of (active) microsclerotia present in the soil.

In this study, growing the different green manure crops or potato followed by incorporating the crop residues was not effective in reducing Verticillium wilt of cauliflower. Other studies reported a significant reduction of Verticillium wilt on potato after green manure treatments [17,35,36]. However, disease control was not consistent throughout these studies reflecting varying conditions and circumstances under which the experiments were conducted. Obviously, factors such as soil type, pathogen inoculum densities, composition of the soil microbiome, and the green manure species used influence the final effect of green manure treatments.

The differences in disease pressure of Verticillium wilt on cauliflower observed between plots could not only be explained by differences in *Verticillium* soil densities. This might be partially attributed to the lack of discrimination between living and dead material, or active and dormant propagules when using a qPCR method to study soil fungi. Hence, the detection of non-viable *Verticillium* microsclerotia, resulting in an overestimation of the soil inoculum, cannot be ignored. It is, however, generally assumed that DNA derived from dead cells degrades fairly rapidly in natural moist soil due to microbial activity, suggesting that the bias due to detection of dead microsclerotia might be of less importance [37,38,39]. Nonetheless, detection of DNA of dormant *Verticillium* microsclerotia is inevitable. Besides the *Verticillium* density, disease pressure is also influenced by other factors such as weather conditions, soil characteristics and other microorganisms present in the soil. For example, it was reported that the incidence of Verticillium wilt on potato was negatively correlated to soil organic matter [40]. A study of Wiggens and Kinkel showed that potatoes grown in soils with greater proportions of antagonistic streptomycetes had lower Verticillium wilt ratings [17].

During the field experiment in which the application of *V. isaacii* Vt305 was tested in four cover crop systems, treating the cauliflower seeds with *V. isaacii* Vt305 was essential to decrease disease severity and incidence. No significant reduction of Verticillium wilt in cauliflower was obtained by growing the different green manure crops without treatment of the seeds with *V. isaacii* Vt305. However, the green manure crops ryegrass, Phacelia, and black oat did not negatively affect the biocontrol effect of *V. isaacii* Vt305 towards Verticillium wilt in cauliflower either. The compatibility of cropping these green manure crops with the application of *V. isaacii* Vt305 in cauliflower is an important result regarding the use of this biocontrol agent in practice to control Verticillium wilt.

Additional field trials to test green manure treatments during two or more successive years, would be useful to assess the long-term effect of green manure crops on soil populations of *Verticillium* and the potential impact on Verticillium wilt of cauliflower. In addition, monitoring other microorganisms during these studies by high-throughput sequencing could be helpful in understanding the effect of green manure crops on Verticillium wilt in cauliflower. Exploration of the diversity of *V. isaacii* populations in our soils will generate more knowledge about the proportion of antagonistic isolates in these populations and thus the potential of increasing the indigenous *V. isaacii* soil population as a strategy to suppress Verticillium wilt of cauliflower. Better insight in the diversity of the indigenous *V. isaacii* soil population is also necessary to estimate the risk of stimulating pathogenic *V. isaacii* isolates by cultivating specific crops and green manures. Recent studies have shown that *V. isaacii* possesses a wide range of ecological lifestyles including pathogenic and endophytic isolates [41,42,43].

The results of this work demonstrated that *V. isaacii* Vt305 has potential to be developed as a biocontrol product to control Verticillium wilt in cauliflower. Furthermore, our results showed that combining the treatment of cauliflower seeds with *V. isaacii* Vt305 and the use of green manure crops were compatible. Although green manure crops alone were not effective in reducing Verticillium wilt in cauliflower in the short term, they did not have a negative effect on the biocontrol effect of *V. isaacii* Vt305. Moreover, green manure crops are generally known to improve soil health and are therefore interesting to integrate in a sustainable and environmentally sound management strategy of Verticillium wilt.

## 4. Materials and Methods

### 4.1. Verticillium isaacii Isolate Vt305 and Inoculum Preparation

*Verticillium isaacii* Vt305 was isolated from soil of a cauliflower field suppressive to Verticillium wilt in Ardooie, Belgium [9].

Conidial suspension was prepared from plates of two-week-old cultures incubated on PDA at room temperature in the dark, by adding sterile distilled water to the plate and gently rubbing the surface of the colony with a sterile spatula. Finally, the conidial suspension was filtered through a sterile cheesecloth and adjusted to the required concentration.

Microsclerotia of *V. isaacii* Vt305 were produced according to the method described by López-Escudero et al. [44] with modifications. Briefly, 0.8 mL conidial suspension (10^6^ conidia mL^−1^) was plated onto each modified soil extract agar plate covered with a sterilized permeable cellophane disc. After 14 days of incubation at room temperature in the dark, microsclerotia were formed on the cellophane sheet. Microsclerotia were scraped off the cellophane with a sterile scalpel, air-dried for several hours in sterile conditions and ground with mortar and pestle to a fine powder. To determine the concentration of the microsclerotia powder, a mix of 3 mg of the powder in 30 mL of sterile water was prepared. The number of microsclerotia in the suspension was then determined in small subsamples (20 µL) using a dissecting microscope and converted to the number of microsclerotia present per mg of powder. Final microsclerotia suspensions were prepared prior to application by mixing a specific amount of the microsclerotia powder with water (sterile distilled water in greenhouse experiments and tap water in field experiments).

### 4.2. Efficacy of V. isaacii Vt305 in Greenhouse Conditions

Two experiments were carried out to determine the efficacy of *V. isaacii* Vt305 in greenhouse conditions in which two different inoculum types, spores or microsclerotia, were tested. In both greenhouse experiments cauliflower (*Brassica oleracea* var *botrytis* L.) plants of the cultivar Cornell were grown in blocks of potting soil for four weeks prior to transplanting to soil naturally infested with *V. longisporum*. When spores were used to inoculate plants with *V. isaacii* Vt305, soil blocks of four-week-old plants were dipped in a conidia suspension with a concentration of 10^6^ conidia mL^−1^ for 30 min. Soil blocks of the control plants were dipped in sterile distilled water water. Ten days after inoculation, each plant together with its soil block was transplanted to a pot (12 L) filled with soil naturally infested with *V. longisporum*. When plants were inoculated with *V. isaacii* Vt305 via microsclerotia, each cauliflower seed was dripped with 0.5 mL of a microsclerotia (MS) suspension using a pipette. Concentrations of 100, 500, and 1000 MS per seed were used. Seeds of the control plants were dripped with sterile distilled water. Thirty days after sowing and inoculation, cauliflower plants were transplanted to pots (12 L) filled with naturally infested soil. The soil used in both experiments originated from a grower’s field in Oppuurs with a history of Verticillium wilt in cauliflower. This soil was sandy loam, pH-KCl 6.8 and 1.8% organic carbon. Analysis of the used soil showed the presence of *V. longisporum* (2974 fg DNA g^−1^ soil) and *V. isaacii* (245 fg DNA g^−1^ soil). Plants were grown at 16–18 °C and 16 h of light. During the experiment in which plants were inoculated with *V. isaacii* Vt305 via spores, six plants per treatment were sampled 28 days post transplantation (dpt) to naturally infested soil to quantify the colonization of the hypocotyl by *V. longisporum* via real-time PCR. In case of inoculation with *V. isaacii* Vt305 via microsclerotia, six plants of each treatment were sampled prior to transplantation to verify the colonization by *V. isaacii* Vt305 of the hypocotyl. To quantify the colonization of the hypocotyl by *V. longisporum* and *V. isaacii* during the experiment, six plants per treatment were sampled at 33, 68, and 103 dpt. Vascular discoloration of 12 plants per treatment was evaluated at respectively 70 dpt and 103 dpt, in the pot experiment with inoculation via spores and the pot experiment with inoculation via microsclerotia.

### 4.3. Efficacy of V. isaacii Vt305 in Field Conditions

Field trials were conducted in grower’s fields located at Ardooie (West Flanders), Bornem and Oppuurs (Antwerp) in 2013, 2014, and 2015. Cauliflower has been cultivated frequently in these fields and Verticillium wilt has been observed. For each trial, the soil characteristics of the field, the amount of soil inoculum of *V. longisporum* and *V. isaacii* present in the field, the inoculation method, the inoculum concentration of *V. isaacii* Vt305, the cauliflower cultivar used, and the planting and harvest date of the cauliflower plants are shown in Table 4. In all field experiments, cauliflower plants were inoculated with *V. isaacii* Vt305 via seed treatment with a microsclerotia suspension. For the field experiments in 2014 and 2015, cauliflower seeds were placed in blocks of potting soil and manually dripped with 0.5 mL tap water (control) or 0.5 mL of microsclerotia suspension of *V. isaacii* Vt305 using a pipette. Different concentrations were used: 300 MS, 500 MS, or 1000 MS per seed. The PHYTO-DRIP^®^ system (Syngenta, Basel, Switzerland) was used to inoculate cauliflower seeds in the field experiment conducted in 2013. During the seeding process in the nursery, a single drop of microsclerotia suspension was dripped onto each seed resulting in a dose of 360 MS of *V. isaacii* Vt305 per seed. Seeds of the control plants were dripped with tap water. Uncoated seeds of the cauliflower cultivars Clapton, Korlanu, and Clarina were used in the field experiments of 2014 and 2015. During the PHYTO-DRIP^®^ field experiment in 2013, uncoated seeds or seeds with two different conventional coatings (coating of iprodione and thiram or coating of fipronil, iprodione, thiram, and metalaxyl-M) of the cauliflower cultivar Clapton were used. Plants were grown for four weeks in the nurseries and subsequently planted in the field. Treatments were arranged in a randomized complete block design with four replicates in the field experiments conducted in 2014 and 2015. Plot sizes were 14.5 m^2^ (4 rows of 9 plants) in Ardooie, 20 m^2^ (6 rows of 8 plants) in Bornem and 20 m^2^ (6 rows of 8 plants) in Oppuurs. During the PHYTO-DRIP^®^ field experiment (2013), plants of each treatment were arranged in four rows of 15 plants in plots of 22.5 m^2^ (one plot per treatment). In each plot, vascular discoloration was scored in stems of 10 (field experiment 2013) or 20 (field experiments 2014 and 2015) randomly chosen plants after harvest.

### 4.4. Colonization of Crop Residues by V. isaacii, V. longisporum and V. dahliae

The green manure crops Phacelia (cv Natra), ryegrass (cv Danergo), sticky nightshade (cv Pion), and black oat (cv Pratex) were sown in boxes (60 cm × 40 cm × 24 cm) filled with soil naturally infested with *Verticillium* spp. (977 fg *V. isaacii* DNA g^−1^ soil, 680 fg *V. longisporum* DNA g^−1^ soil and 996 fg *V. dahliae* DNA g^−1^ soil). The soil originated from a grower’s field located in Ardooie (sandy loam, pH-KCl 5.7 and 0.9% organic carbon). Each green manure crop was sown in one box at the sowing density indicated by the seed company. Four months after sowing, plants were removed from the soil (shoot and root) and cut into small fragments of ±2 cm. For each crop, three nylon bags (5 cm × 5 cm) were filled with the plant fragments and buried at a depth of 10 cm for three months in the soil of the same box in which the crop had grown. Boxes were regularly watered during the experiment and placed in a non-heated greenhouse compartment.

### 4.5. Effect of Potato and Green Manure Crops on Soil Population of Verticillium and the Impact on Verticillium Wilt in Cauliflower in Field Conditions

Four field trials were carried out at Puurs (Antwerp) and Ardooie (West Flanders) in 2013 and 2014 regarding the effect of potato and green manure crops on *Verticillium* soil populations and the impact on Verticillium wilt in cauliflower. Table 5 shows the location, the soil characteristics, the cultivated crops preceding cauliflower (green manure crops and potato), and the timing of the cultivation practices for each of the experiments. The same field was used in Ardooie for the consecutive experiments in 2013 and 2014, while in Puurs the experiments were carried out in two different fields. The plot sizes in the experiments in Puurs were respectively 20 m^2^ and 40 m^2^ in 2013 and 2014. In the field located in Ardooie, each plot had a size of 67.5 m^2^. Treatments were arranged in a randomized complete block design with four replicates. Green manure crops and potato were incorporated in the soil before planting of the cauliflower plantlets in the plots. The cauliflower cultivar Clarina was used in all field experiments, except in the experiment of Puurs in 2013 where the cultivar Clapton was planted. To monitor the inoculum densities of *V. isaacii*, *V. longisporum* and *V. dahliae*, soil samples were collected at three time points: one week before sowing the green manure crops/potato, one week before the incorporation of the green manure crops/potato, and one week before harvest of the cauliflower heads.

### 4.6. Combined Effect of Green Manure Crops and Treatment of the Cauliflower Seeds with V. isaacii Vt305 on Verticillium Wilt of Cauliflower

The effect of combining green manure crops and treatment of the cauliflower seeds with *V. isaacii* Vt305 on Verticillium wilt of cauliflower was tested during a field experiment in 2015 at Puurs. The location and soil characteristics of the field, the cultivated green manure crops preceding cauliflower, and the timing of the cultivation practices are shown in Table 5. The experimental design was a four-by-two factorial combination of four cover crop systems (fallow, Phacelia, black oat, and ryegrass) and two main crop systems (untreated cauliflower plants and cauliflower plants inoculated with *V. isaacii* Vt305). Treatments were arranged in a randomized complete block design with four replicates. Plot sizes in the field in Puurs were 35 m^2^. Green manure crops were incorporated in the soil before planting of the cauliflower plantlets in the plots. To monitor the inoculum densities of *V. isaacii*, *V. longisporum*, and *V. dahliae*, soil samples were collected at three time points: one week before sowing the green manure crops/potato, one week before the incorporation of the green manure crops/potato, and one week before harvest of the cauliflower heads.

### 4.7. Disease Assessment

Stems of cauliflower were longitudinally split to score vascular discoloration caused by *V. longisporum*. A scale from 0 to 3 was used, where 0 = no vascular discoloration; 1 = vascular discoloration of <50% of stem length; 2 = vascular discoloration of 50–75% of stem length; and 3 = vascular discoloration of 76–100% of stem length. The disease index was calculated based on the scores for vascular discoloration via the formula of Townsend–Heuberger [(0*a + 1*b + 2*c + 3*d)/(e*f)]*100; letters a, b, c, and d, are the numbers of plants for each disease score, the letter e is the highest score (=3), and f represents the number of observations. The quality of the harvested curds was evaluated and expressed as percentage of curds that meet the quality requirements of the market.

### 4.8. DNA Extraction from Plant Tissues

In the greenhouse experiments regarding the efficacy of *V. isaacii* Vt305, the roots and the hypocotyl (below the cotyledons) were sampled to quantify the colonization by *Verticillium* spp. In case of the colonization experiment of the crop residues, the contents of three bags were pooled and carefully rinsed with tap water to remove the residual soil prior to DNA extraction. Plant samples were ground with mortar and pestle in liquid nitrogen and DNA was extracted using the Invisorb Spin Plant Mini DNA extraction kit (Invitek). A Nanodrop spectrophotometer was used to measure DNA concentrations and DNA extracts were diluted to a final concentration of 5 ng µL^−1^ prior to real-time PCR analysis.

### 4.9. Soil Sampling and Processing to Determine the Inoculum Density

Twelve soil cores were collected to a depth of 30 cm from each plot and bulked into a single soil sample per plot. Samples were air dried at room temperature for two weeks. According to the method described by Debode et al. [45], fifty grams of air-dried soil were crumbled and thoroughly mixed with 50 mL 70% (*wt*/*wt*) sucrose. After centrifuging the suspension at 2700× *g* for 20 min, the resulting supernatant was poured into a vacuum-filtration system (Gelman Sciences, Port Washington, NY, USA) over a 20-µm nylon mesh filter (Millipore, Burlington, MA, USA) and rinsed with sterile water. DNA was extracted from the material retained on the filter, using the MoBio Power Soil DNA isolation kit following manufacturer’s instructions. DNA concentrations were measured using a Nanodrop spectrophotometer and DNA extracts were diluted to a final concentration of 5 ng µL^−1^ for analysis via real-time PCR.

### 4.10. Real-Time PCR Analysis

Real-time PCR was performed in reactions of 50 µL containing 5 µL template, 300 nM of the *V. isaacii* primers or 200 nM of the *V. longisporum*/*V. dahliae* primers, and 25 µL GoTaq qPCR Master Mix containing the CXR reference dye (Promega) to monitor dsDNA synthesis. The primers used to detect and quantify the different *Verticillium* spp. in the soil and plant samples are listed in Table 6. Amplification and melting curve analysis were performed using the MX3005P real-time PCR Detection System (Stratagene). The thermal profile consisted of 10 min at 95 °C, followed by 40 cycles of 15 s at 95 °C and 1 min at 60 °C. Melting curves were obtained by heating the samples to 95 °C for 15 s, cooling to 60 °C for 15 s and heating again to 95 °C for 15 s to verify specific amplification. Quantification was done using the standard curve technique with a 10-fold dilution series of DNA in sterile water (10–10^6^ fg DNA per reaction) from reference strains Ve005 (*V. dahliae*) (lab collection), VLO1 (*V. longisporum*) [2] and Vt305 (*V. isaacii*) [9].

### 4.11. Statistical Analysis

All data of the experiments were analyzed using the software package SPSS 26.0 for windows. The ordinal data of vascular discoloration were analyzed using non-parametric Mann–Whitney comparisons (*p* < 0.05). Data on the colonization by *V. longisporum* and *V. isaacii* and data on the inoculum densities of *Verticillium* spp. were ln transformed before statistics and further analyzed using Tukey tests (*p* < 0.05). Data on the disease index, the disease incidence and the percentage marketable curds did not meet the conditions of normality and homogeneity of variances and were analyzed using non-parametric Mann–Whitney comparisons (*p* < 0.05).

## Figures and Tables

**Table 1:** Biocontrol of Verticillium wilt of cauliflower by *V. isaacii* Vt305 in field conditions at three locations (Ardooie and Bornem in 2014 and Oppuurs in 2015). Cauliflower seeds (cv Clapton, Korlanu or Clarina) were treated with water (control) or a microsclerotia (MS) suspension of *V. isaacii* Vt305 at different concentrations: 300 MS, 500 MS, or 1000 MS per seed. At harvest (76 dpt in Ardooie, 73 dpt in Bornem and 66 dpt in Oppuurs), vascular discoloration of the stems was scored: 0 = no vascular discoloration; 1 = vascular discoloration of <50% of stem length; 2 = vascular discoloration of 50–75% of stem length; and 3 = vascular discoloration of 76–100% of stem length. For each location the percentage of plants with a specific score for vascular discoloration (mean of 4 plots ± standard error), the disease index (%) (mean of 4 plots ± standard error) and the percentage of marketable cauliflower curds are shown.

	Cultivar	% Plants	% Plants	% Plants	% Plants	Disease	Marketable
		Score 0	Score 1	Score 2	Score 3	Index (%) ^1^	Curds (%)
**Ardooie**							
Control	Clapton	4.4 ± 2.2 a ^2^	22.2 ± 2.2 a	48.9 ± 4.4 a	24.5 ± 2.2 a	64.4 ± 2.2 a	75.5 ± 2.2 a
300 MS	Clapton	17.8 ± 8.0 b	37.8 ± 4.5 ab	33.3 ± 11.5 a	11.1 ± 4.4 ab	45.9 ± 7.4 b	88.9 ± 4.4 b
500 MS	Clapton	11.1 ± 2.2 ab	42.2 ± 5.9 b	46.7 ± 7.7 a	0.0 ± 0.0 c	45.2 ± 3.2 b	100.0 ± 0.0 c
1000 MS	Clapton	13.3 ± 3.8 ab	48.9 ± 8.9 b	35.6 ± 4.4 a	2.2 ± 2.2 bc	42.2 ± 2.2 b	97.8 ± 2.2 c
**Bornem**							
Control	Korlanu	48.0 ± 10.6 a	45.0 ± 8.5 a	7.0 ± 2.5 a	0.0 ± 0.0 a	19.7 ± 4.3 a	100.0 ± 0.0 a
300 MS	Korlanu	50.0 ± 10.4 a	45.0 ± 5.7 a	5.0 ± 5.0 ab	0.0 ± 0.0 a	18.3 ± 5.1 a	100.0 ± 0.0 a
500 MS	Korlanu	72.0 ± 6.9 b	27.0 ± 6.0 b	1.0 ± 1.0 b	0.0 ± 0.0 a	9.7 ± 2.6 a	100.0 ± 0.0 a
1000 MS	Korlanu	57.0 ± 27.0 a	40.0 ± 12.0 ab	3.0 ± 1.9 ab	0.0 ± 0.0 a	15.3 ± 5.0 a	100.0 ± 0.0 a
**Oppuurs**							
Control	Korlanu	10.7 ± 6.3 ab	39.3 ± 8.1 a	36.9 ± 7.1 a	13.1 ± 4.9 a	50.8 ± 3.7 a	86.9 ± 4.9 a
500 MS	Korlanu	9.6 ± 5.1 a	57.1 ± 3.9 b	21.4 ± 5.0 b	11.9 ± 7.1 a	45.2 ± 6.0 ab	88.1 ± 7.1 a
Control	Clarina	15.5 ± 9.0 ab	48.8 ± 3.0 ab	35.7 ± 10.4 a	0.0 ± 0.0 b	40.1 ± 6.4 b	100.0 ± 0.0 b
500 MS	Clarina	21.4 ± 12.4 b	60.7 ± 7.6 b	15.5 ± 4.1 b	2.4 ± 1.4 b	32.9 ± 6.2 c	97.6 ± 1.4 b

^1^ The disease index was calculated based on the scores for vascular discoloration via the formula of Townsend-Heuberger [(0*a + 1*b + 2*c + 3*d)/(e*f)]*100; letters a, b, c, and d, are the numbers of plants for each disease score, the letter e is the highest score (=3), and f represent the number of observations (= 20). ^2^ For each location, values with the same letter within the same column do not differ significantly according to the Mann-Whitney U-test (*p* < 0.05).

**Table 2:** Biocontrol of Verticillium wilt of cauliflower in field conditions at Ardooie in 2013 when the PHYTO-DRIP^®^ system was used to deliver microsclerotia of *V. isaacii* Vt305 on seeds. Cauliflower seeds (cv Clapton) with coatings (seed coating 1 = iprodione and thiram; seed coating 2 = fipronil, iprodione, thiram and metalaxyl-M) or without coating were treated with water (control) or a suspension of *V. isaacii* Vt305 microsclerotia (MS) at a concentration of 360 MS per seed using the PHYTO-DRIP^®^ system. At harvest (98 dpt), vascular discoloration of the stems was scored: 0 = no vascular discoloration; 1 = vascular discoloration of < 50% of stem length; 2 = vascular discoloration of 50–75% of stem length; and 3 = vascular discoloration of 76–100% of stem length. For each treatment the percentage of plants (*n* = 10) with a specific score for vascular discoloration, the disease index (%) and the disease incidence (%) are shown.

Seed	Seed	% Plants	% Plants	% Plants	% Plants	Disease	Marketable
Treatment	Coating	Score 0	Score 1	Score 2	Score 3	Index (%) ^1^	Curds (%)
Water	No coating	0.0 a ^2^	0.0 a	90.0 a	10.0 a	70.0 a	90.0 a
Water	Coating 1	0.0 a	20.0 ab	60.0 a	20.0 a	66.7 a	80.0 a
Water	Coating 2	0.0 a	0.0 a	90.0 a	10.0 a	70.0 a	90.0 a
Vt305	No coating	20.0 a	70.0 c	10.0 b	0.0 a	30.0 b	100.0 a
Vt305	Coating 1	10.0 a	90.0 c	0.0 b	0.0 a	30.0 b	100.0 a
Vt305	Coating 2	30.0 a	60.0 bc	10.0 b	0.0 a	26.7 b	100.0 a

^1^ The disease index was calculated based on the scores for vascular discoloration via the formula of Townsend–Heuberger [(0*a + 1*b + 2*c + 3*d)/(e*f)]*100; letters a, b, c, and d, are the numbers of plants for each disease score, the letter e is the highest score (=3), and f represents the number of observations (= 10). ^2^ Values with the same letter within the same column do not differ significant according to the Mann–Whitney U-test (*p* < 0.05).

**Table 3:** Colonization of the green manure residues by *Verticillium* spp. Green manures were grown for four months in soil naturally infested with *V. isaacii, V. longisporum*, and *V. dahliae*, subsequently cut into small pieces (shoot and roots) and put in nylon bags that were buried in the soil for three months. The colonization of these plant residues by the three *Verticillium* species was quantified via real time-PCR. Samples of three bags were pooled before real time-PCR analysis and values are the detected amounts of *Verticillium* DNA in these pooled samples.

Crop Residue	*V. isaacii* DNA (fg DNA mg^−1^ Plant Tissue)	*V. longisporum* DNA (fg DNA mg^−1^ Plant Tissue)	*V. dahliae* DNA (fg DNA mg^−1^ Plant Tissue)
Phacelia	673	-	1304
Ryegrass	75,644	-	1531
Sticky nightshade	7405	-	102,809
Black oat	106	80	944

**Table 4:** Overview of the field trials in which the effect of artificial inoculation of cauliflower plants with *V. isaacii* Vt305 on Verticillium wilt was tested. For each experiment, the location, the soil characteristics, the amount of soil inoculum of *V. longisporum* (Vl) and *V. isaacii* (Vi), the inoculation method and inoculum concentration of *V. isaacii* Vt305 (Vt305), the cauliflower cultivar used, and the planting and harvest date of the cauliflower plants are shown.

	Trial 1	Trial 2	Trial 3	Trial 4
**Location**	Ardooie	Bornem	Oppuurs	Ardooie
**Geographic coordinates field**	50.95799, 3.18380	51.09646, 4.29868	51.06842, 4.24148	50.95799, 3.18380
**Year**	2014	2014	2015	2013
**Soil type**	Sandy loam	Sandy loam	Sandy loam	Sandy loam
**Soil pH-KCl**	5.7	6.5	6.8	5.7
**Soil % C_org_**	0.9	1.6	1.8	0.9
**Vl soil inoculum** (fg DNA g^−1^ soil)	1941	358	1749	231
**Vi soil inoculum** (fg DNA g^−1^ soil)	268	127	290	232
**Inoculation method Vt305**	Drip (pipette): seed	Drip (pipette): seed	Drip (pipette): seed	PHYTO-DRIP^®^: seed
**Inoculum concentration Vt305**	300, 500 and 1000 MS/plant	300, 500 and 1000 MS/plant	500 MS/plant	360 MS/plant
**Cauliflower cultivar**	Clapton	Korlanu	Korlanu Clarina	Clapton ^1^
**Cauliflower planting**	05/08	25/07	22/05	31/07
**Cauliflower harvest**	20/10	06/10	27/07	06/11

MS: microsclerotia. ^1^ Uncoated seeds, seeds coated with iprodione and thiram and seeds coated with fipronil, iprodione, thiram, and metalaxyl-M were used.

**Table 5:** Overview of the field trials carried out in 2013, 2014 and 2015 regarding the effect of potato and green manure crops on *Verticillium* soil population and the impact on Verticillium wilt. The location, the soil characteristics, the cultivated crops preceding cauliflower (green manure crops and potato), and the timing of the cultivation practices are shown.

	Trial 1	Trial 2	Trial 3	Trial 4	Trial 5
**Location**	Puurs	Ardooie	Puurs	Ardooie	Puurs
**Geographic coordinates field**	51.06851, 4.25162	50.95799, 3.18380	51.06753, 4.25180	50.95799, 3.18380	51.06851, 4.25162
**Year**	2013	2013	2014	2014	2015
**Soil type**	Sandy loam	Sandy loam	Sandy loam	Sandy loam	Sandy loam
**Soil pH-KCl**	6.9	5.7	7.3	5.7	6.9
**Soil % C_org_**	1.9	0.9	2.2	0.9	1.9
**Green manures ^1^/potato ^2^**	Ryegrass Phacelia Sticky nightshade Potato	Ryegrass Phacelia Sticky nightshade Potato	Ryegrass Phacelia Black oat Potato	Ryegrass Phacelia Black oat Potato	Ryegrass Phacelia Black oat
**Sowing green manures/planting potato**	18/04	16/04	11/03	17/04	18/03
**Incorporation green manures/potato**	16/07	28/07	11/06	14/07	02/06
**Cauliflower planting ^3,4^**	30/07	30/07	07/07	05/08	17/06
**Cauliflower harvest**	04/11	23/10	01/10	03/11	14/09

^1^ Ryegrass (*Lolium multiflorum* Lam.) cv Danergo: 60 kg/ha; Phacelia (*Phacelia tanacetifolia* Benth.) cv Natra: 10 kg/ha; Sticky nightshade (*Solanum sisymbriifolium* Lam.) cv Pion: 3 kg/ha; Black oat (*Avena strigosa* Schreb.) cv Pratex 50 kg/ha. ^2^ Potato (*Solanum tuberosum* L.) cv Anosta (Puurs and Ardooie) in 2013, and cv Monalisa (Puurs) and cv Première (Ardooie) in 2014: 35,714 tubers/ha. ^3^ The cauliflower cultivar Clapton was planted in Puurs in 2013 and in all the other field trials the cauliflower cultivar Clarina was planted. ^4^ In field trial 5 (Puurs 2015) cauliflower seeds were treated with a microsclerotia suspension of *V. isaacii* Vt305 (500 MS per seed) or tap water (control).

**Table 6:** Primers used to detect and quantify *Verticillium* spp. in plant and soil samples.

Target Organism	Gene Target	Primer	Sequence (5′ to 3′)	Reference
*V. isaacii*	rDNA ITS	VtF4	CCGGTGTTGGGGATCTACT	[45]
		VtR2	GTAGGGGGTTTAGAGGCTG	[45]
*V. longisporum*	18S intron rDNA	VlspF1	AGCCTGAGTCACGAGAGATATGGG	[46]
		VlspR4	CAAACCACGCCACTGCATTCTCGT	[46]
*V. dahliae*	rDNA ITS	VdF1	CCGCCGGTCCATCAGTCTCTCTG	[46]
		VdR1	GGGACTCCGATGCGAGCTGTAAC	[46]
